# Effectiveness of Non-nucleoside Reverse-Transcriptase Inhibitor-Based Antiretroviral Therapy in Women Previously Exposed to a Single Intrapartum Dose of Nevirapine: A Multi-country, Prospective Cohort Study

**DOI:** 10.1371/journal.pmed.1000233

**Published:** 2010-02-16

**Authors:** Jeffrey S. A. Stringer, Michelle S. McConnell, James Kiarie, Omotayo Bolu, Thanomsak Anekthananon, Tavatchai Jariyasethpong, Dara Potter, Winnie Mutsotso, Craig B. Borkowf, Dorothy Mbori-Ngacha, Peter Muiruri, John Odero Ong'ech, Isaac Zulu, Lungowe Njobvu, Bongkoch Jetsawang, Sonal Pathak, Marc Bulterys, Nathan Shaffer, Paul J. Weidle

**Affiliations:** 1 University of Alabama at Birmingham Centre for Infectious Disease Research in Zambia, Lusaka, Zambia; 2 Thailand Ministry of Public Health—US Centers for Disease Control and Prevention (CDC) Collaboration, Nonthaburi, Thailand; 3 Global AIDS Program, CDC, Atlanta, Georgia, United States of America; 4 Kenyatta National Hospital, Nairobi, Kenya; 5 University of Nairobi, Nairobi, Kenya; 6 Siriraj Hospital, Mahidol University, Bangkok, Thailand; 7 Rajavithi Hospital, Bangkok, Thailand; 8 Global AIDS Program, CDC-Zambia, Lusaka, Zambia; 9 Global AIDS Program, CDC-Kenya, Nairobi, Kenya; 10 Division of HIV/AIDS Prevention, CDC, Atlanta, Georgia, United States of America; 11 University Teaching Hospital, Lusaka, Zambia; 12 Northrup-Grumman Corporation, Atlanta, Georgia, United States of America; National Institute of Child Health and Human Development, United States of America

## Abstract

**Background:**

Intrapartum and neonatal single-dose nevirapine (NVP) reduces the risk of mother-to-child HIV transmission but also induces viral resistance to non-nucleoside reverse transcriptase inhibitor (NNRTI) drugs. This drug resistance largely fades over time. We hypothesized that women with a prior single-dose NVP exposure would have no more than a 10% higher cumulative prevalence of failure of their NNRTI-containing antiretroviral therapy (ART) over the first 48 wk of therapy than would women without a prior exposure.

**Methods and Findings:**

We enrolled 355 NVP-exposed and 523 NVP-unexposed women at two sites in Zambia, one site in Kenya, and two sites in Thailand into a prospective, non-inferiority cohort study and followed them for 48 wk on ART. Those who died, discontinued NNRTI-containing ART, or had a plasma viral load ≥400 copies/ml at either the 24 wk or 48 wk study visits and confirmed on repeat testing were characterized as having failed therapy. Overall, 114 of 355 NVP-exposed women (32.1%) and 132 of 523 NVP-unexposed women (25.2%) met criteria for treatment failure. The difference in failure rates between the exposure groups was 6.9% (95% confidence interval [CI] 0.8%–13.0%). The failure rates of women stratified by our predefined exposure interval categories were as follows: 47 of 116 women in whom less than 6 mo elapsed between exposure and starting ART failed therapy (40%; *p*<0.001 compared to unexposed women); 25 of 67 women in whom 7–12 mo elapsed between exposure and starting ART failed therapy (37%; *p* = 0.04 compared to unexposed women); and 42 of 172 women in whom more than 12 mo elapsed between exposure and starting ART failed therapy (24%; *p* = 0.82 compared to unexposed women). Locally weighted regression analysis also indicated a clear inverse relationship between virologic failure and the exposure interval.

**Conclusions:**

Prior exposure to single-dose NVP was associated with an increased risk of treatment failure; however, this risk seems largely confined to women with a more recent exposure. Women requiring ART within 12 mo of NVP exposure should not be prescribed an NNRTI-containing regimen as first-line therapy.

*Please see later in the article for the Editors' Summary*

## Abstract 2

In a comparative cohort study, Jeffrey Stringer and colleagues investigate the risk of ART failure in women who received single-dose nevirapine for PMTCT, and assess the duration of increased risk.

## Editors' Summary

**Background:**

Every year, acquired immunodeficiency syndrome (AIDS) kills nearly 300,000 children. At the end of 2008, 2.1 million children were positive for the human immunodeficiency virus (HIV), the cause of AIDS, and in that year alone more than 400,000 children were newly infected with HIV. Most HIV-positive children acquire the virus from their mothers during pregnancy or birth or through breastfeeding, so-called mother-to-child transmission (MTCT). Without intervention, 15%–30% of babies born to HIV-positive women become infected with HIV during pregnancy and delivery, and a further 5%–20% become infected through breastfeeding. These rates of infection can be greatly reduced by treating the mother and her newborn baby with antiretroviral drugs. A single dose of nevirapine (a “non-nucleoside reverse transcriptase inhibitor” or NNRTI) given to the mother at the start of labor and to the baby soon after birth reduces the risk of MTCT by nearly a half; a further reduction in risk can be achieved by giving the mother and her baby additional antiretroviral drugs during pregnancy, around the time of birth, and while breast-feeding.

**Why Was This Study Done?:**

Single-dose nevirapine is the mainstay of MTCT prevention programs in many poor countries but can induce resistance to nevirapine and to other NNRTIs. The drugs used to treat HIV infections fall into several different classes defined by how they stop viral growth. HIV can become resistant to any of these drugs and a virus strain that is resistant to one member of a drug class is often also resistant to other members of the same class. Because most first-line antiretroviral therapies (ARTs; cocktails of antiretroviral drugs) used in developing countries contain an NNRTI and because HIV-positive mothers eventually need ART to safeguard their own health, the resistance to NNRTIs that is induced in women by single-dose nevirapine might decrease the chances that ART will work for them later. In this multi-country, prospective cohort study, the researchers compare the effectiveness of NNRTI-containing ART in a group (cohort) of women previously exposed to single-dose nevirapine during childbirth to its effectiveness in a group of unexposed women. They also investigate whether the length of time between nevirapine exposure and ART initiation affects ART effectiveness.

**What Did the Researchers Do and Find?:**

The researchers enrolled 355 HIV-positive nevirapine-exposed women and 523 HIV-positive nevirapine-unexposed women in Zambia, Kenya, and Thailand who were just starting NNRTI-containing ART and followed them for 48 weeks. They defined ART failure as death, discontinuation of NNRTI-containing ART, or a high virus load in the blood (virologic failure) at 24 or 48 weeks. ART failed in nearly a third of the nevirapine-exposed women but in only a quarter of the nevirapine-unexposed women. Women who began ART within 6 months of taking single-dose nevirapine to prevent MTCT were twice as likely to experience ART failure as women not exposed to single-dose nevirapine. Women who began ART 7–12 months after single-dose nevirapine had a slightly increased risk of ART failure compared to unexposed women but this increased risk was not statistically significant; that is, it could have occurred by chance. Women who began ART more than 12 months after single-dose nevirapine did not have an increased risk of ART failure compared to unexposed women. Finally, the researchers used a statistical method called locally weighted regression analysis to confirm that an increase in the interval between single-dose nevirapine and ART initiation decreased the risk of virologic failure.

**What Do These Findings Mean?:**

These findings, which confirm and extend the results of previous studies and which are likely to be generalizable to other resource-poor countries, indicate that single-dose nevirapine given to women to prevent MTCT increases their risk of subsequent ART failure. More positively, they also show that this increased failure risk is largely confined to women who begin ART within a year of exposure to nevirapine. Because of the study design, it is possible that the nevirapine-exposed women share some additional, undefined characteristic that makes them more likely to fail ART than unexposed women. Even so, these findings suggest that, provided NNRTI-containing ART is not given to HIV-positive women within a year of nevirapine exposure, single-dose nevirapine can be safely used to prevent MTCT without compromising the mother's future antiretroviral treatment options.

**Additional Information:**

Please access these Web sites via the online version of this summary at http://dx.doi.org/10.1371/journal.pmed.1000233.

- Information is available from the US National Institute of Allergy and Infectious Diseases on HIV infection and AIDS, on treatments for HIV/AIDS, and on HIV infection in infants and children
- HIV InSite has comprehensive information on all aspects of HIV/AIDS
- Information is available from Avert, an international AIDS charity, on many aspects of HIV/AIDS, including information on children, HIV, and AIDS and on preventing mother-to-child transmission of HIV (in English and Spanish)
- UNICEF also has information about children and HIV and AIDS (in several languages)
- The World Health Organization has information on mother-to-child transmission of HIV

## Introduction

In many parts of the world, pediatric AIDS remains an uncontrolled epidemic [1]. The vast majority of children who become HIV infected acquire the virus from their mothers, either during pregnancy or delivery, or afterward, while breast-feeding [2]. Intrapartum and neonatal single-dose nevirapine (NVP) can reduce the risk of perinatal HIV transmission by nearly half [3]. This efficacy is maintained even in the face of breast-feeding [4] and can be enhanced through the addition of antenatal zidovudine (ZDV) [5] or combination ZDV/lamivudine (3TC) [6]. The operational simplicity of single-dose NVP has made it the cornerstone intervention for prevention of mother-to-child HIV transmission (PMTCT) worldwide [7]-[10], yet its convenience may come at an unintended cost. One-third or more of women who are exposed to the drug—either alone or as a component of a non-suppressive prophylactic regimen—will develop high-level resistance to the non-nucleoside reverse transcriptase inhibitor (NNRTI) class of antiretroviral drugs [11],[12]. Population genotyping methods indicate that these drug resistance mutations (which peak in prevalence within 8 wk of exposure) gradually fade over a period of 6 to 12 months [13],[14]. However, results from more sensitive assays suggest that drug resistance mutations may persist indefinitely in minority virus populations [15]. The clinical implications of this resistance for women who eventually need antiretroviral therapy (ART) for their own health are not completely understood, but are concerning [16],[17], given the worldwide predominance of NNRTI-containing regimens for first-line ART.

We sought to evaluate the effectiveness of NNRTI-based ART in women who were previously exposed to a single, intrapartum dose of NVP. We were particularly interested in the effect of the interval between NVP exposure and ART inception, and whether there was any clear temporal threshold for increased risk of virologic failure.

## Methods

### Study Population

The NNRTI Response Study enrolled single-dose NVP-exposed and -unexposed women starting ART at two sites in Lusaka, Zambia (Kanyama Health Centre and Matero Reference Centre), one site in Nairobi, Kenya (Kenyatta National Hospital), and two sites in Bangkok, Thailand (Siriraj and Rajavithi Hospitals). Study patients were treated according to national protocols operant in each country. All participating sites were public-sector outpatient clinics with ongoing, free ART services available. Women who were at least 18 y of age and who met local criteria to start ART were eligible to participate. In Kenya and Zambia, women who met any of the following criteria were eligible: (1) CD4^+^ cell count <200/µl; (2) World Health Organization (WHO) clinical stage IV; or (3) WHO stage III and CD4^+^ cell count <350/µl. In Thailand, women whose CD4^+^ cell count was <200/µl or who had clinical AIDS were eligible. In all countries, we excluded from the study women who were currently pregnant, those with any prior exposure to ART (other than single-dose NVP and/or ZDV monotherapy for PMTCT), and those who were not starting an NNRTI-based first-line therapy.

We determined NVP exposure through a structured procedure that considered the dates of a prospective enrollee's last pregnancy in relation to available PMTCT services at the delivery site and reviewed her prior labor and delivery records, if available. We also accepted verbal confirmation of prior single-dose NVP ingestion, provided the woman was able to identify a NVP tablet from a photograph. In cases where NVP exposure status was uncertain, we did not offer entry into the study.

The date of exposure was taken as the date of delivery. In the few women who reported more than one prior exposure to single-dose NVP, we took the date of the most recent exposure. During protocol planning, we noted that NVP-unexposed women available for study recruitment comprised mostly self-referrals of women with symptomatic HIV disease; they were thus generally more ill upon presentation than NVP-exposed women, who tended to be referred from postnatal care. In an effort to mitigate the effect of confounding by disease status on our study outcomes, we frequency-matched NVP-exposed and unexposed women by both CD4^+^ cell count and WHO stage at entry. Our matching scheme used a 3×3 table that included three CD4^+^ strata (0–49 cells/µl, 50–200 cells/µl, and >200 cells/µl) and three WHO stage strata (WHO stage I/II, III, and IV). We originally planned to enroll equal numbers of NVP-exposed and -unexposed women in each cell of this 3×3 table; however, in June 2006, in response to a slower than expected rate of enrollment among NVP-exposed women, we increased the enrollment of unexposed to exposed women to a 3∶2 ratio.

### Clinical Care and Study Procedures

In each of the three countries, most clinical care was delivered by non-physician clinicians (i.e., nurses and/or clinical officers), with rotating study physicians providing oversight. The study did not direct individual treatment decisions, but rather relied upon local guidelines and clinician discretion to manage patients. We attempted to evaluate all NVP-exposed women for study enrollment. Because unexposed women were much more numerous, each site enrolled only a subset of these unexposed women, according to availability of open cells within our disease-status matching scheme. At study enrollment, study clinicians conducted a history and physical examination, a clinical evaluation for tuberculosis (TB) co-infection, and additional laboratory tests including hemoglobin concentration and plasma viral load. Each site quantified HIV-1 RNA in venous plasma using a commercially available polymerase chain reaction assay. The Siriraj Hospital in Bangkok used the COBAS TaqMan HIV-1 Test; all other sites used the Roche Amplicor HIV-1 Monitor v. 1.5 (both from Roche Molecular Systems, Pleasanton, CA, USA).

First-line ART drug regimens were started within 2 wk of study enrollment and included two nucleoside reverse transcriptase inhibitors (3TC, plus either stavudine [d4T] or ZDV), and one NNRTI (either NVP or efavirenz [EFV]) [18]. In Kenya and Zambia, we used only proprietary drug formulations; in Thailand, we used a locally produced fixed-dose combination of d4T/3TC/NVP for most women (GPO-VIR). In Kenya and Thailand, all patients except one started a d4T-containing regimen, whereas in Zambia patients started either ZDV or d4T according to drug availability (ZDV was avoided in patients whose baseline hemoglobin was <10 g/dl). To avoid a drug interaction between NVP and rifampin, clinicians generally deferred ART (and study enrollment) for patients receiving acute phase therapy for tuberculosis, unless their CD4^+^ lymphocyte count was <50 cells/µl, in which case ART was started immediately with an EFV-based regimen. Each site prescribed co-trimoxazole prophylaxis against *Pneumocystis* pneumonia to women who met WHO criteria for its use [19].

Study visits occurred once prior to starting ART, once on or around the day of ART commencement, then at weeks 2, 4, 8, 16, 24, 36, and 48 on ART. Routine clinical and pharmacy visits occurred according to local guidelines and varied only slightly. The Zambian and Kenyan sites dispensed antiretroviral drugs in monthly increments, whereas the Thai sites dispensed according to the same schedule as study visits.

We repeated the CD4^+^ cell count and plasma viral load tests at 24 and 48 wk. Women who had ≥400 copies of viral RNA/ml plasma at either of these scheduled visits were evaluated for medication adherence and scheduled for a repeat viral load 1 mo later. Those who had persistent viremia were categorized as virologic failures and the clinicians caring for them had the option of switching to second-line therapy with a ritonavir-boosted protease inhibitor or continuing NNRTI-based ART. The study protocol did not direct individual treatment decisions, but, in general, clinician discretion in the management of low-level viremia (e.g., <1,000 copies/ml) was such that, in some cases, women who were clinically and immunologically well were managed expectantly and not immediately switched to second-line therapy. Those who had <400 copies of viral RNA/ml plasma on repeat measurement were considered responders, and a third repeat measurement not routinely performed.

Patients on ART presented at scheduled intervals to the clinic to collect their antiretroviral drugs. Each dispensation included a 2- or 3-d buffer of extra pills, and we allowed pre-registered treatment partners to collect a patient's medication. Missed visits were followed up by home visits and/or telephone calls. Nurses or pharmacy technicians performed adherence counseling. We assessed adherence by the participants' report of the number of doses missed in the 7 d before each scheduled visit, and a priori, chose to dichotomize self-reported adherence as ≥95% or <95%.

### Study Design and Definition of Failure

The NNRTI Response Study was designed as a prospective, observational, non-inferiority study [20]. The primary study hypothesis was that women who had previously received single-dose NVP for PMTCT would have a failure rate on NNRTI-based ART that was not more than 10% greater than that observed for women without a prior exposure (i.e., a non-inferiority margin of 10%). Under this non-inferiority design, the failure rate for the NVP-exposed group would be considered equivalent to that of the NVP-unexposed group if the upper bound of the 95% confidence interval (CI) for the difference between groups did not exceed 10%. The study was also designed to compare the failure rate among unexposed women to that of (a) women exposed to NVP within 6 mo of starting ART, (b) women exposed 7–12 mo before starting ART, and (c) women exposed >12 mo prior to starting ART. The primary study outcome was assessed at 48 wk after initiating ART. For the primary analysis, a participant was considered as having failed at 48 wk if she died prior to that time, was no longer receiving NNRTI-based ART for any reason, or had a plasma viral load ≥400 copies/ml (confirmed with repeat testing) at either the 24 or 48 wk study visits.

We also conducted two planned secondary analyses of treatment failure, namely (a) one that excluded women who died within 90 d of starting therapy, and (b) an “on-treatment” analysis that excluded women who died, were lost to follow-up, or switched to second-line therapy for reasons of toxicity. In contrast to the primary analysis, the on-treatment analysis did not categorize as failures those women whose viral load was ≥400 copies/ml at 24 wk who subsequently went on to achieve suppression at 48 wk, unless they were switched to a second-line therapy by their clinician in response to the 24 wk viral load measurement.

For the sample size calculation for the primary analysis, we assumed a 20% failure rate in the NVP-unexposed group and chose a non-inferiority margin of 10%. Thus, if the failure rate in the exposed group was <30%, we would consider that rate non-inferior. We assumed a failure rate of 20% in the NVP-exposed group and a 15% drop-out rate. Under these assumptions we would need 235 women in each group to have 80% power, or 350 women in each group (700 total) to have 90% power, to establish non-inferiority using an asymptotic normal test of difference to compare two independent binomial proportions at the one-sided alpha = 0.05 significance level. As the difference between the two failure rates increases, however, the sample size required to establish non-inferiority also increases.

### Statistical Analyses

We present standard summary statistics of the enrollment or baseline data, including counts, proportions, medians, and interquartile ranges (IQRs). When appropriate, variables were categorized using standard or clinically relevant cut-points. Nonparametric Wilcoxon rank-sum tests and Kruskall-Wallace tests were used to compare the medians of selected baseline variables between countries and between NVP-exposed and unexposed women. We generated a histogram to illustrate the distribution of the intervals between single-dose NVP exposure and ART commencement (the “exposure interval”). We calculated exact binomial confidence intervals around the proportion of treatment failures for NVP unexposed women and for each of the three exposure interval categories and used Fisher's exact test to compare these proportions. We used exact logistic regression to estimate the odds of treatment failure as a function of individual baseline variables. For the multiple logistic regression analyses, we included in all models a common set of baseline predictor variables (exposure interval, country, CD4^+^ count, viral load, WHO stage, and age). We also included hemoglobin and body mass index in particular models when they were significant at the *p*<0.05 level (Wald Chi-square test). We then performed multiple logistic regression to model the odds of treatment failure as a function of selected baseline variables and to estimate adjusted odds ratios (ORs) with Wald confidence intervals [21]. Furthermore, we performed locally weighted regression (using the LOESS procedure in SAS software) to model nonparametrically the risk of treatment failure as a function of the exposure interval for the primary and on-treatment analyses [22]. All of these statistical calculations were performed using SAS software, version 9.1.3 (SAS Institute, Cary, North Carolina).

### Study Monitoring

The study protocol and its informed consent procedures were approved before study initiation by the Institutional Review Boards at the Thai Ministry of Public Health, the Mahidol University - Siriraj Hospital, the University of Zambia School of Medicine, the Kenyatta National Hospital (Kenya), University of Alabama at Birmingham and US Centers for Disease Control and Prevention (CDC). All participants provided written informed consent. A Data Monitoring Committee with representatives from each country and from CDC, who were not involved in the study, met periodically to review study progress and interim analyses. Enrollment began on 9 June 2005 in Zambia, on 15 August 2005 in Thailand, and on 9 June 2006 in Kenya. We completed enrollment at all sites the week of 22 January 2007. All women were followed for 48 wk.

## Results

### Cohort Description

**Figure 1:**
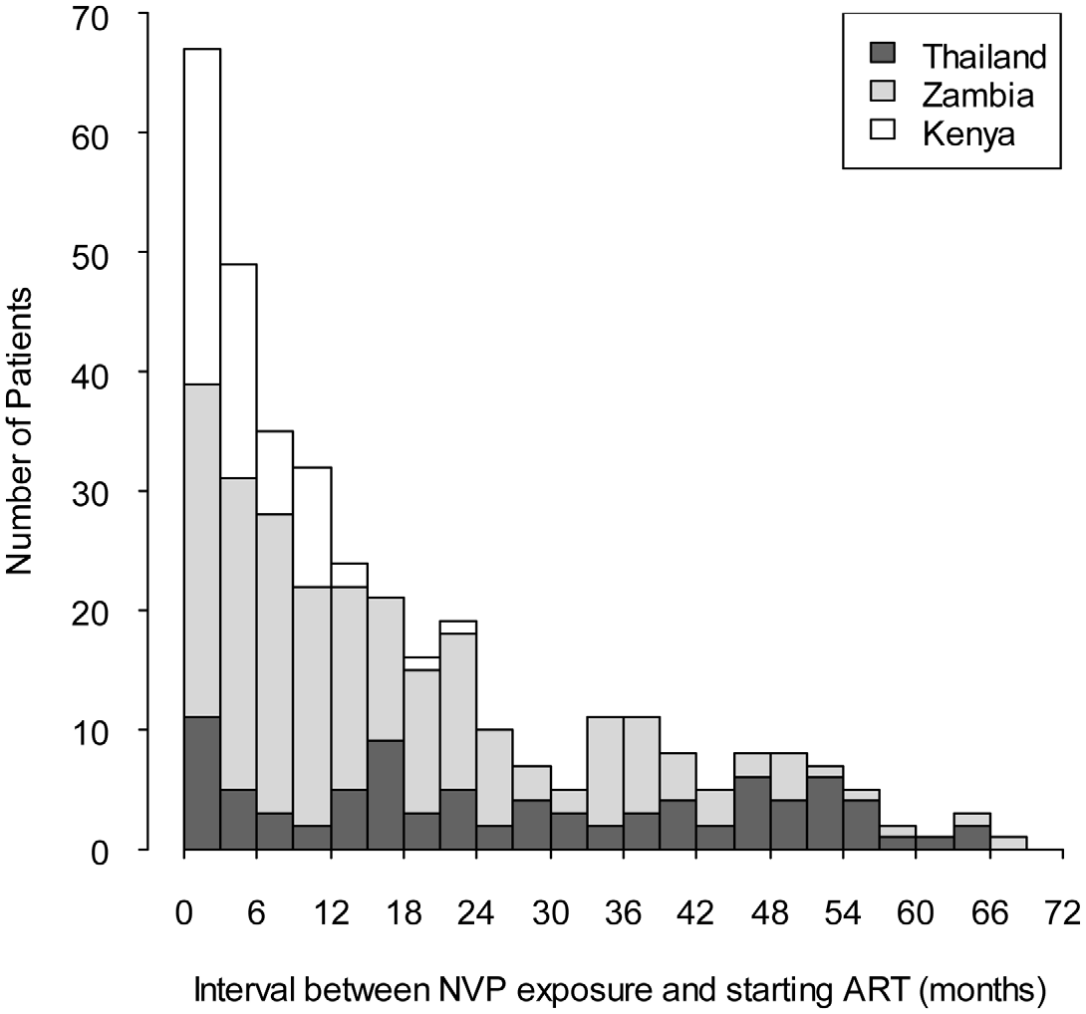
Interval between exposure to single-dose nevirapine and starting NNRTI-based antiretroviral therapy in the NNRTI Response Study—Zambia, Kenya, Thailand (2005 – 2008).

We enrolled 878 women, 509 (58%) from Zambia, 152 (17%) from Kenya, and 217 (25%) from Thailand (Table 1). Compared to Zambian women, Kenyan women weighed more (median 55 versus 52 kg, *p*<0.05; Wilcoxon rank-sum test) and had lower median hemoglobin concentrations (median 10.4 versus 10.8 g/dl, *p*<0.05), while Thai women had higher body-mass index (median 21.1 versus 19.7 kg/m^2^) and higher hemoglobin concentrations (median 11.4 versus 10.8 g/dl, *p*<0.05). A total of 355 women (40%) were NVP-exposed and 523 (60%) were NVP-unexposed (Table 2). NVP-exposed women were younger (29 versus 33 median y, *p*<0.05), had higher median CD4^+^ counts (160 versus 139 cells/µl; *p*<0.05), and had lower median plasma viral loads at baseline (5.0 versus 5.2 log copies/ml, *p*<0.05). The median time between NVP ingestion and starting ART among the 355 exposed women was 11.8 mo (IQR 4.4–24.7) and differed by country: in Kenya, the median interval was 3.9 mo (IQR 1.8–7.2), in Zambia it was 12.0 mo (IQR 5.7–23.1), and in Thailand it was 24.2 mo (IQR 12.3–45.9; *p*<0.05; Kruskall-Wallace test; Figure 1). All women in Kenya and Zambia received single-dose NVP only. All women in Thailand received short-course ZDV in addition to single-dose NVP for PMTCT. No women received an antiretroviral therapy “tail” in order to limit emergence of NVP resistance after delivery, because that practice had not been implemented as standard of care in any of these countries at the time of single-dose NVP exposure by these women.

**Table 1:** Baseline characteristics of participants enrolled in the NNRTI Response Study—Zambia, Kenya, Thailand (2005–2008).

Characteristics	Zambia (*n* = 509)		Kenya (*n* = 152)		Thailand (*n* = 217)	
	*n* (%)	Median (IQR)	*n* (%)	Median (IQR)	*n* (%)	Median (IQR)
Age, y		32 (28–36)		32 (28–36)		32 (28–37)
<30	189 (37)		54 (36)		75 (35)	
Weight, kg		52 (46–57)		55 (47.8–60.8)*		50 (45–56)
<50	195 (38)		46 (30)		104 (48)	
Body mass index, kg/m^2^		19.7 (18.3–21.9)		19.9 (17.7–22.6)		21.1 (18.8–23.2)*
<18	113 (22)		49 (32)		38 (18)	
WHO stage						
I or II	215 (42)		61 (40)		140 (64)	
III	254 (50)		70 (46)		34 (16)	
IV	40 (8)		21 (14)		43 (20)	
CD4^+^ lymphocyte count, cells/µl		148 (88–211)		147 (76.5–212.5)		148 (50–220)
0 to 49	63 (12)		27 (18)		54 (25)	
50 to 199	304 (60)		80 (53)		95 (44)	
≥200	142 (28)		45 (30)		68 (31)	
Plasma viral load, log copies/ml ([missing = 6)		5.0 (4.4–5.4)		5.3 (4.8–5.9)		5.0 (4.6–5.5)
<4.0	74 (15)		15 (10)		25 (12)	
4.0 to <5.0	186 (37)		31 (20)		79 (36)	
≥5.0	243 (48)		106 (70)		113 (52)	
Hemoglobin, g/dl (missing = 16)		10.8 (9.6–12.0)		10.4 (8.8–12.0)*		11.4 (10.4–12.3)*
<8.0	33 (7)		15 (10)		6 (3)	
NNRTI initially prescribed						
NVP	497 (98)		131 (86)		192 (88)	
EFV	12 (2)		21 (14)		25 (12)	
TB at baseline	44 (9)		12 (8)		15 (7)	
Interval between nevirapine exposure and starting NNRTI-based ART (exposed women only), mo	*N* = 201	12.0 (5.7–23.1)	*N* = 67	3.9 (1.8–7.2)*	*N* = 87	24.2 (12.3–45.9)*
Unexposed	308 (61)		85 (56)		130 (60)	
1–6	54 (11)		46 (30)		16 (7)	
7–12	46 (9)		16 (11)		5 (2)	
>12	101 (20)		5 (3)		66 (30)	

*p-Value<0.05, compared to Zambia by Wilcoxon rank-sum test.

**Table 2:** Baseline characteristics of participants enrolled by prior exposure to single-dose nevirapine in the NNRTI Response Study—Zambia, Kenya, Thailand (2005–2008).

Characteristics	Unexposed to Single-Dose Nevirapine (*N* = 523)		Exposed to Single-Dose Nevirapine (*N* = 355)		Total	
	*n* (%)	Median (IQR)	*n* (%)	Median (IQR)	*n* (%)	Median (IQR)
Age, y		33 (29–39)		29 (26–33)*		32 (28–36)
<30	136 (26)		182 (51)		318 (36)	
Weight, kg		52 (47–58)		51 (45–58)		52 (46–58)
<50	200 (38)		145 (41)		345 (39)	
Body mass index, kg/m^2^		20.2 (18.4–22.6)		19.7 (18.0–22.3)		20.0 (18.3–22.5)
<18	111 (21)		89 (25)		200 (23)	
WHO stage						
I or II	236 (45)		180 (51)		416 (47)	
III	219 (42)		139 (39)		358 (41)	
IV	68 (13)		36 (10)		104 (12)	
CD4+ lymphocyte count, cells/µl		139 (71–209)		160 (90–219)*		147.5 (77–215)
0 to 49	98 (19)		46 (13)		144 (16)	
50 to 199	280 (54)		199 (56)		479 (55)	
≥200	145 (28)		110 (31)		255 (29)	
Plasma viral load, log copies/ml [missing = 6]		5.2 (4.6–5.6)		5.0 (4.3–5.4)*		5.1 (4.5–5.5)
<4.0	52 (10)		62 (17)		114 (13)	
4.0 to <5.0	179 (34)		117 (33)		296 (34)	
≥5.0	288 (55)		174 (49)		462 (53)	
Hemoglobin, g/dl (missing = 16)		10.8 (9.5–12.0)		11.0 (9.8–12.2)		10.9 (9.6–12.1)
<8.0	31 (6)		23 (7)		54 (6)	
NNRTI initially prescribed						
NVP	481 (91)		339 (95)		820 (93)	
EFV	42 (8)		16 (5)		58 (7)	
TB at baseline	53 (10)		18 (5)		71 (8)	

*p-Value<0.05, compared to unexposed by Wilcoxon rank-sum test.

### Patient Status at 48 Weeks of Follow-Up

**Figure 2:**
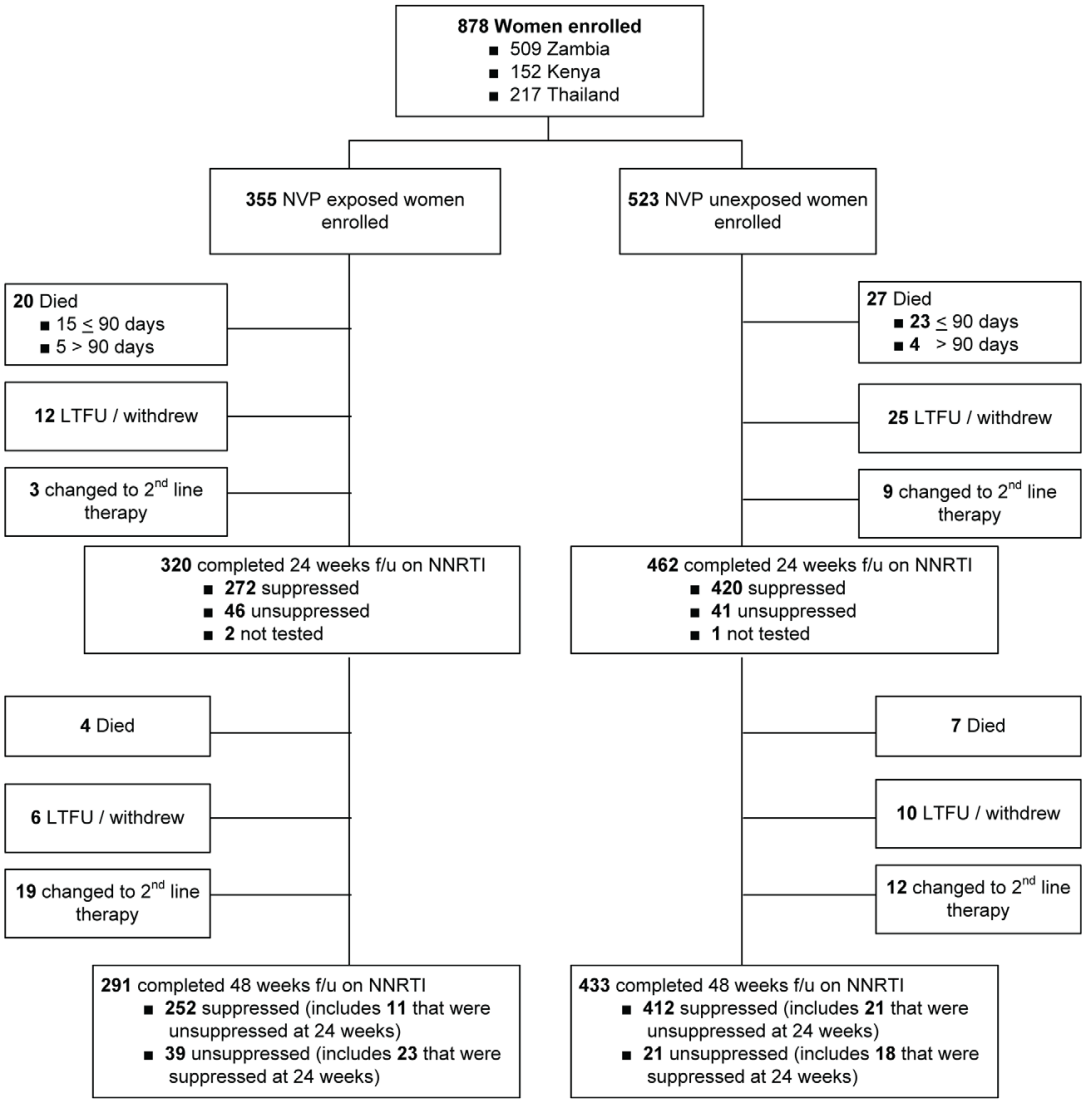
Study Schema for the NNRTI Response Study—Zambia, Kenya, Thailand (2005–2008). Among the 779 women who completed 24 wk follow-up on NNRTI-based ART (excluding the three women who were temporarily off therapy), self-reported adherence over the five visits by week 24 was greater than 95% for 440 (95%) of 461 NVP-unexposed women and 300 (94%) of 318 NVP-exposed women (*p* = 0.5). Among the 724 women who completed 48 weeks on NNRTI-based ART, self-reported adherence over the two visits at weeks 36 and 48 was greater than 95% for 419 (97%) of 433 NVP-unexposed women and 280 (96%) of 291 NVP-exposed women (*p* = 0.7). f/u, follow-up; LTFU, lost to follow-up.

Between enrollment and 48 wk of follow-up, 58 women (6.6%) died, including 38 (4.3%) before 90 d on therapy (Figure 2). Fifty-seven of 58 deaths involved circumstances that were probably or possibly related to HIV disease, and one death was attributed to a road traffic accident. An additional 53 women (6.0%) were lost to follow-up or voluntarily withdrew from the study. Forty-three women (4.9%) were switched by their provider to a non-NNRTI-based antiretroviral regimen: 29 women (3.3%) were switched after a 24-wk viral load measurement indicated virologic failure, 12 women (1.4%) were switched for reasons of toxicity before 24 wk, and two women (<1%) were switched for reasons of toxicity between 25 and 48 wk. In total, 724 women (82%) completed 48 wk of follow-up on an NNRTI-containing regimen.

**Figure 3:**
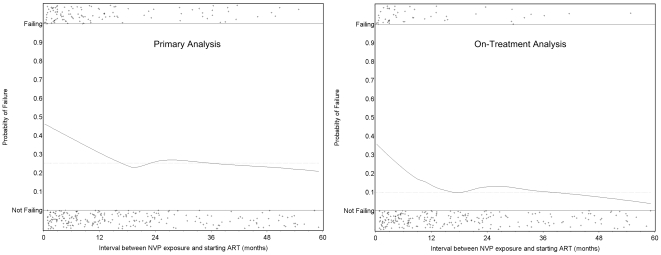
Time between exposure to single-dose NVP and starting antiretroviral therapy and the probability of treatment failure in the NNRTI Response Study—Zambia, Kenya, Thailand (2005–2008). Locally weighted regression (LOESS) models of the risk of treatment failure as a function of the time interval between NVP ingestion and starting ART. The left panel defines treatment failure according to the study's primary definition; the right panel defines treatment failure according to a planned secondary definition (see Methods). The horizontal dotted line indicates the failure rate among the women who were not exposed to single-dose NVP. The individual plusses (+) indicated on each panel represent individual patients who were either failing (top) or not failing (bottom).

NVP-exposed women were more likely to meet the study's primary criteria for failure at 48 wk than were NVP-unexposed women (114 of 355 [32.1%; 95% CI 27.2%–37.2%] versus 132 of 523 [25.2%; 95% CI 21.6%–29.2%]). The difference in failure rates between the NVP-exposed and unexposed groups was 6.9% (95% CI 0.8%–13.0%). The upper bound of this 95% CI exceeds our predefined 10% non-inferiority margin (i.e., we failed to confirm non-inferiority). The treatment failure rates of women stratified by exposure interval were as follows: 47 of 116 women in whom less than 6 mo elapsed between exposure and starting ART failed therapy (41%; 95% CI 32%–50%; *p* = 0.001 compared to unexposed women, Fisher's exact test); 25 of 67 women in whom 7–12 mo elapsed between exposure and starting ART failed therapy (37%; 95% CI 26%–50%; *p* = 0.04 compared to unexposed women, Fisher's exact test); and 42 of 172 women in whom more than 12 mo elapsed between exposure and starting ART experienced treatment failure (24%; 95% CI 18%–32%; *p* = 0.92 compared to unexposed women, Fisher's exact test). This relationship was also evident in locally weighted regression analysis, which suggests the increased risk of treatment failure may extend to women in whom as many as 15 mo have elapsed between NVP ingestion and starting ART (Figure 3).

### Factors Associated with Treatment Failure at 48 Weeks in the Primary Analysis

In both crude and adjusted analysis, women who had an interval of less than 6 mo between exposure to NVP and starting ART were more likely to experience treatment failure than women without exposure (adjusted OR 2.16; 95% CI 1.34–3.49; Table 3). Women whose exposure interval was between 7 and 12 mo had an increased risk of treatment failure that was not statistically significant (adjusted OR 1.47; 95% CI 0.82–2.65), whereas women whose exposure interval was more than 12 mo had no evidence of increased risk (adjusted OR 1.02; 95% CI 0.66–1.59). Also associated with failure in crude and adjusted analyses were enrollment at a Kenyan or Zambian site, baseline CD4^+^ count 0–49 cells/µl, plasma viral load ≥5.0 log copies/ml, WHO stage III or IV disease, age <30 y, hemoglobin <8.0 g/dl, and body mass index <18 kg/m^2^ (Table 3). Thirty-eight women died before completing 90 d of ART, including 23 (4.4%) of 523 unexposed women and 15 (4.2%) of 355 NVP-exposed women (*p* = 0.9, Fisher's exact test). Excluding these early deaths from the primary analysis did not appreciably change the findings (Table 4).

**Table 3:** Factors associated with treatment failure in the primary analysis in the NNRTI Response Study—Zambia, Kenya, Thailand (2005–2008).

Factors	No. Failures/*N* (%)	Unadjusted ORs (95% Exact Confidence Limits)	Adjusted ORs^a^ (95% Wald Confidence Limits)
Interval between single-dose nevirapine exposure and starting NNRTI-based ART, mo			
Unexposed	132/523 (25)	Ref	Ref
1-6	47/116 (41)	2.02 (1.29, 3.13)	2.16 (1.34, 3.49)
7–12	25/67 (37)	1.76 (0.99, 3.09)	1.47 (0.82, 2.65)
>12	42/172 (24)	0.96 (0.63, 1.45)	1.02 (0.66, 1.59)
Country			
Thailand	34/217 (16)	Ref	Ref
Zambia	159/509 (31)	2.44 (1.60, 3.81)	2.60 (1.63, 4.14)
Kenya	53/152 (35)	2.87 (1.71, 4.89)	2.17 (1.23, 3.82)
CD4^+^ lymphocyte count (cells/µl)			
≥200	58/255 (23)	Ref	Ref
50 to 199	126/479 (26)	1.21 (0.84, 1.77)	1.21 (0.81, 1.81)
0 to 49	62/144 (43)	2.56 (1.61, 4.09)	2.60 (1.56, 4.33)
Plasma viral load, log copies/ml (missing = 6)			
<4.0	20/114 (17)	Ref	Ref
4.0 to <5.0	75/296 (25)	1.59 (0.90, 2.92)	1.72 (0.96, 3.08)
≥5.0	149/462 (32)	2.24 (1.31, 3.98)	2.01 (1.14, 3.55)
WHO stage			
I or II	88/416 (21)	Ref	Ref
III	121/358 (34)	1.90 (1.36, 2.66)	1.47 (1.01, 2.13)
IV	37/104 (36)	2.06 (1.25, 3.35)	1.55 (0.91, 2.65)
Age (y)			
≥30	130/560 (23)	Ref	Ref
<30	116/318 (36)	1.90 (1.39, 2.60)	1.81 (1.29, 2.54)
Hemoglobin, g/dl; (missing = 16)			
≥8.0	215/808 (27)	Ref	Ref
<8.0	27/54 (50)	2.75 (1.52, 5.00)	1.94 (1.06, 3.55)
Body mass index, kg/m^2^			
≥18	161/678 (24)	Ref	Ref
<18	85/200 (42)	2.37 (1.68, 3.35)	1.66 (1.15, 2.39)
Weight, kg			
≥50	123/533 (23)	Ref	—
<50	123/345 (36)	1.85 (1.35, 2.52)	—
NNRTI initially prescribed			
NVP	231/820 (28)	Ref	—
EFV	15/58 (26)	0.89 (0.45, 1.67)	—
TB at baseline			
No active TB	221/807 (27)	Ref	—
Active TB	25/71 (35)	1.44 (0.83, 2.46)	—

See Methods for definition of primary analysis.

a Adjusted ORs controlling for country, CD4 cell count, viral load, WHO stage, age, hemoglobin, and body mass index (all as categorical variables as in Table 1. Twenty-one observations are not included, owing to missing baseline results for either viral load or hemoglobin.

Ref, referent group.

**Table 4:** Relationship between exposure interval and treatment failure at 48 weeks in the NNRTI Response Study—Zambia, Kenya, Thailand (2005–2008).

Interval between Single-Dose NVP Exposure and Starting ART	Primary Analysis^a^			Primary Analysis, Excluding Deaths before 90 Days^b^			On-Treatment Analysis^c^		
	No. Failures/*N* (%)	Unadjusted ORs (95% Exact Confidence Limits)	Adjusted ORs (95% Wald Confidence Limits)	No. Failures/*N* (%)	Unadjusted ORs (95% Exact Confidence Limits)	Adjusted ORs (95% Wald Confidence Limits)	No. Failures/*N* (%)	Unadjusted ORs (95% Exact Confidence Limits)	Adjusted ORs^b^ (95% Wald Confidence Limits)
Unexposed	132/523 (25)	Ref	Ref	109/500 (22)	Ref	Ref	38/450 (8)	Ref	Ref
1–6 mo	47/116 (41)	2.02 (1.29, 3.13)	2.16 (1.34, 3.49)	45/114 (39)	2.34 (1.52, 3.60)	2.35 (1.45, 3.82)	33/107 (31)	4.84 (2.85, 8.20)	5.25 (2.87, 9.60)
7–12 mo	25/67 (37)	1.76 (0.99, 3.09)	1.47 (0.82, 2.65)	19/61 (31)	1.62 (0.91, 2.90)	1.42 (0.76, 2.64)	5/51 (10)	1.18 (0.44, 3.14)	1.05 (0.38, 2.88)
>12 mo	42/172 (24)	0.96 (0.63, 1.45)	1.02 (0.66, 1.59)	35/165 (21)	0.97 (0.63, 1.48)	0.98 (0.62, 1.56)	20/152 (13)	1.64 (0.92, 2.92)	1.92 (1.03, 3.58)

a Adjusted ORs controlling for country, CD4 cell count, viral load, WHO stage, age, hemoglobin, and body mass index (all as categorical variables as in table 1). See methods for definition of primary analysis.

b Adjusted ORs controlling for country, CD4 cell count, viral load, WHO stage, and age (all as categorical variables as in table 1) – hemoglobin and body-mass index were not retained in the final model.

c Adjusted ORs controlling for country, CD4 cell count, viral load, WHO stage, and age (all as categorical variables as in table 1) - hemoglobin and body-mass index were not retained in the final model. A separate multivariate model that included self-reported adherence did not alter the adjusted ORs in any appreciable way. See methods for definition of on-treatment analysis.

Ref, reference group.

### Factors Associated with Virologic Failure in the On-Treatment Analysis

At 24 wk of follow-up 41 (9%) of 462 unexposed women and 46 (14%) of 320 NVP-exposed women had a plasma viral load ≥400 copies/ml. Of these 87 women with detectable virus at 24 wk, 31 were changed to a protease inhibitor-based regimen and 56 continued their NNRTI-based therapy. Of 56 whose NNRTI-based therapy was continued, 51 completed 48 wk on therapy, of whom 32 (63%) later achieved viral load suppression to <400 copies/ml. This included 21 (88%) of 24 unexposed women and 11 (41%) of 27 NVP-exposed women (*p*<0.001; Fisher's exact test). Of the 692 women whose viral load was suppressed at 24 wk, 673 completed 48 wk on NNRTI-based therapy, of whom 41 (6%) were no longer suppressed at 48 wk. This included 18 (4%) of 409 unexposed women and 23 (9%) of 264 NVP-exposed women (*p* = 0.02; Fisher's exact test).

In the on-treatment analysis, which was limited to women who completed 48 wk of follow-up on NNRTI-based ART or who were changed to a second-line regimen in response to virologic failure at 24 wk, 38 (8%) of 450 unexposed women met criteria for failure, compared to 33 (31%) of 107 women exposed to NVP ≤6 mo before starting ART (adjusted OR 5.40; 95% CI 2.94–9.92), 5 (10%) of 51 women exposed 7–12 mo prior (adjusted OR 1.02; 95% CI 0.37–2.82), and 20 (13%) of 152 women exposed >12 mo prior (adjusted OR 1.97; 95% CI 1.05–3.68) (Table 4).

### Prior Receipt of Single-Dose NVP in Multiple Pregnancies

Fourteen women (13 Zambian, one Kenyan) received single-dose NVP during more than one pregnancy. Among these women, the median time between the first and the more recent NVP exposure was 1,061 days (IQR 742–1,297). Virologic failure was found in five (50%) of ten women who completed 48 wk in the study, including four (57%) of seven whose most recent exposure was ≤6 mo before starting ART, 0 (0%) of one exposed 7–12 months prior, and one (50%) of two exposed >12 mo prior.

### CD4^+^ Cell Count Response

The median CD4^+^ count among all participants at baseline was 148 cells/µl (IQR 77–215; *n* = 878), at 24 wk was 282 cells/µl (IQR 191–371; *n* = 786), and at 48 wk was 294 cells/µl (IQR 206–404; *n* = 762). The median CD4^+^ cell count change from baseline to 24 wk was 123 cells/µl (IQR, 57–207), and from baseline to 48 wk was 149 cells/µl (IQR 75–232). CD4^+^ response did not differ significantly among women in the four exposure interval strata (unpublished data).

## Discussion

In this large, multi-country, prospective study, women with prior exposure to a single, intrapartum dose of NVP were more likely to meet the study's primary criteria for treatment failure than were women who were not exposed. The overall treatment failure rate at 48 wk—which we defined conservatively to include not only detectable viral load but also death, loss to follow-up, and regimen changes for any reason—was 28% in this cohort, and was associated in multiple logistic regression analysis with a number of factors in addition to prior NVP exposure, including country of enrollment. The increased risk of treatment failure in our study appears to be concentrated in women in whom a short period of time had elapsed between NVP exposure and starting ART. NVP-exposed women in whom this interval was more than 12 mo had essentially the same prevalence of failure at 48 wk as women without prior exposure.

When the definition of treatment failure was narrowed to include only virologic outcomes (the “on treatment” analysis), we observed a similarly increased risk of virologic failure among women with shorter intervals between NVP exposure and starting NNRTI-based ART. In fact, by this secondary definition, the risk of failure was statistically evident even in those women starting NNRTI-based ART in our pre-defined category of 12 mo or more after exposure. It should be noted, however, that locally weighted regression analysis indicates a clear dose-response relationship between exposure interval and virologic failure, and that the increased risk of failure by either our primary or on-treatment failure definition is largely absent by 15 mo.

In 2004, Lallemant and colleagues reported on clinical outcomes of 269 Thai women who initiated NNRTI-based ART following participation in the Perinatal HIV Prevention Trial, where use of single-dose NVP was randomized [15]. At 6 mo after ART initiation, 96 (51%) of 188 NVP-exposed women had >50 virus copies/ml plasma compared to 13 (32%) of 41 women without prior exposure (*p* = 0.03). In their study, the median time between delivery and ART initiation was short (6.1 mo), and the authors did not report comparisons of failure rates based upon interval between NVP exposure and starting ART. In a follow-up report presented in abstract form, the women were followed through 18 mo and no additional failures were observed in either group [23]. Thus, failure attributable to NVP exposure in their study appeared to manifest within the first 6 mo of therapy.

Working in Botswana, Lockman and colleagues in 2007 were the first to report that timing between NVP exposure and ART initiation might be an important predictor of subsequent virologic failure [17]. Like the Thai trial [15], participants in the Botswana study were randomized to single-dose NVP or placebo when they presented in labor. While NVP exposure was associated with increased risk of virologic failure at 6 mo (8.4% versus 5.0%; *p* = 0.002), this risk was mainly attributable to recent exposure, defined as occurring within 6 mo of ART initiation. NVP-exposed women with greater exposure intervals appeared to have no additional risk of failure. This finding is consistent with observational studies in Zimbabwe [24], Côte d'Ivoire [25], Zambia [26],[27], and South Africa [28].

In this study, we also noted that the small group of women not virologically suppressed to <400 copies/ml at 24 wk, but who eventually achieved suppression at 48 wk with continued NNRTI-based therapy, contained a significantly greater proportion of women without prior single-dose NVP compared with those who did not achieve suppression by 48 wk. Although we cannot fully assess whether this finding might have resulted from differences in care between these two groups, it suggests that women with prior NVP exposure who do not achieve virologic suppression at 24 wk have a greater likelihood of remaining unsuppressed at 48 wk. This observation raises the possibility that clinicians should consider switching to second-line therapy for NVP-exposed women who do not achieve viral load suppression at 24 wk on NNRTI-based therapy.

Strengths of our study include its large cohort size, good follow-up rates, high reported adherence to treatment with correspondingly high virologic suppression, and inclusion of women with both short and long intervals between exposure to intrapartum NVP and starting ART. These characteristics of the study allowed a fairly nuanced analysis of the relationship between the exposure interval and risk of future virologic failure (Figure 3). In addition, the study's multi-country population and differing approaches to clinical HIV management support the external validity of our conclusions. The primary limitation of our study is that the exposure of interest—use of intrapartum NVP—was not randomized, leaving open the possibility that exposed and unexposed women may differ in some systematic way other than their single-dose NVP use. During the protocol planning process, we noted that NVP-unexposed women seeking care tended to have more advanced HIV disease than did exposed women. Even among women of similar CD4^+^ status, NVP-unexposed women had generally more advanced disease by WHO staging than did NVP-exposed women. Thus, we elected to group-match our enrollees on both CD4^+^ and WHO disease stage to mitigate this potential confounding. We decided not to use ultrasensitive viral load testing for patient management nor to categorize failure in our analysis for two reasons. First, the more sensitive viral load assay was not available at the African sites and would have required expatriation of study specimens, a practice that is increasingly discouraged by local authorities. Second, the less sensitive assay is the current standard for clinical practice in both African sites. We thus believed that use of a 400 copies/ml threshold would yield outcomes that reflected the realities of local clinical practice. Third, we followed women for 48 wk, and longer-term virologic response rates were not assessed. Finally, our results may not apply to women who receive a prophylactic “tail” of other drugs, such as ZDV/3TC [29] or single-dose tenofovir/emtricitabine [30] in order to reduce the emergence of single-dose NVP-related resistance.

Ever since the HIVNET 012 trial demonstrated its prophylactic efficacy [3], intrapartum NVP has been a nexus of controversy [31]–[33]. Perhaps the strongest opposition to single-dose NVP surrounds its potential to induce viral resistance and thereby adversely affect a mother's future treatment options. Fortunately, this report, along with others [5],[17],[24]–[27], provides some reassurance (Table 5). It is now evident that the risk of intrapartum NVP compromising a mother's future response to an NNRTI-containing regimen is confined mostly to women in whom ART will be needed within 12–15 mo of delivery. Where CD4^+^ testing is routinely available, programs could minimize the number of women who fall into this risk group by increasing the CD4^+^ threshold for starting ART in pregnancy to 350 cells/µl, as recommended in the 2006 WHO guidelines [29]. The benefit of this approach would be 2-fold. First, it would ensure that most women exposed to single-dose NVP would not need therapy for at least a year. Second, through the provision of suppressive therapy to precisely those women at highest transmission risk, it would prevent more perinatal HIV infections [29]. In the occasional circumstance where a woman did need therapy soon after single-dose NVP exposure, a protease inhibitor-containing regimen or a triple nucleoside regimen could be prescribed [34].

**Table 5:** Studies of virologic response rates to NNRTI-based treatment among women previously exposed to single-dose NVP.

Study Site [Reference]	Follow-Up Reported in This Table^a^	Viral Load Threshold for Treatment Response, Copies/ml	PMTCT Regimen^b^	Exposure Interval	*n* c	Proportion Responding to Treatment^d^	*p*-Value (versus SDNVP Unexposed)
Thailand [16]	6 mo	<50	ZDV	—	41	68%	
			ZDV + SDNVP	Median 6 mo since exposure	188	49%	0.03
Botswana [17]	12 mo	<400	ZDV	<6 mo since delivery	36	97%	
			ZDV + SDNVP	<6 mo since exposure	24	54%	<0.001
			ZDV	≥6 mo since delivery	70	86%	
			ZDV + SDNVP	≥6 mo since exposure	88	88%	0.76
Zimbabwe [24]	12 mo	<400	ZDV		32	72%	
			SDNVP	Median 17 mo since exposure	20	65%	0.60
Zambia [26]	6 mo	<400	Unexposed		0		
			SDNVP	<6 mo since exposure	8	38%	—
			SDNVP	6–12 mo since exposure	22	59%	—
			SDNVP	12–24 mo since exposure	61	72%	—
			SDNVP	>24 mo since exposure	70	77%	—
Cote d'Ivoire [27]	12 mo	<500	Unexposed		97	85%	
			SDNVP ± ZDV or ZDV/3TC e	Median 21 mo since exposure	122	78%	0.23
South Africa [28]	6 mo	<50	Unexposed		60	91%	
			SDNVP	18–36 mo since exposure	94	98%	0.21
Zambia, Kenya, Thailand [this study]	12 mo	<400	Unexposed		450	92%	—
			SDNVP ± ZDV^f^	<6 mo since exposure	107	69%	<0.01
			SDNVP ± ZDV^f^	7–12 mo since exposure	51	90%	0.79
			SDNVP ± ZDV^f^	>12 mo since exposure	152	87%	0.11

a Some studies report outcomes at additional time points that are not listed here.

b Women who received ZDV or ZDV/3TC typically received the regimen from ∼36 wk gestation.

c Numbers of patients includes those from whom a virologic response was available in the report.

d Proportions estimated by Kaplan-Meier method for references [17] and [28]. All others are raw proportions.

e Women who received NVP for PMTCT in this study got SDNVP alone, ZDV + SDNVP, or ZDV/3TC + SDNVP. Response rates listed here are from Table 3 in reference [27]. *p*-Value calculated using Fisher's exact test.

f All women in Thailand who were exposed to SDNVP also received antenatal ZDV; in Zambia and Kenya antenatal ZDV was not used. *p*-Values calculated using Fisher's exact test for each exposure interval compared to SDNVP unexposed.

SDNVP, single dose intrapartum nevirapine.

Worldwide, approximately one-third of HIV-exposed infants receive perinatal HIV prophylaxis in any form [35]. Single-dose intrapartum and neonatal NVP is among the simplest and most feasible interventions available and remains a cornerstone of perinatal HIV prevention in many under-resourced settings. This study indicates that, when used judiciously in conjunction with ART for women eligible for treatment, single-dose NVP to prevent mother-to-child HIV transmission can be administered without substantially comprising the mother's future antiretroviral treatment options.

## Abbreviations

3TC: combination ZDV/lamivudine
ART: antiretroviral therapy
CI: confidence interval
EFV: efavirenz
IQR: interquartile range
NNRTI: non-nucleoside reverse transcriptase inhibitor
NVP: nevirapine
OR: odds ratio
PMTCT: prevention of mother-to-child HIV transmission
d4T: stavudine
TB: tuberculosis
WHO: World Health Organization
ZDV: zidovudine
